# Supplementary material for: Molecular Testing as Triage in Cervical Cancer Screening: Economic Evaluation Using Headroom Analysis
Source: Cancers (Basel). 2025 Feb 11;17(4):612. doi: 10.3390/cancers17040612 (PMC11853484; doi:10.3390/cancers17040612)
Supplement: Supplementary file 1 [file cancers-17-00612-s001.zip › cancers-3417124-supplementary.pdf]

**Supplementary Material**

**Model Development**

## 1. Summary

The microsimulation Markov model for cervical cancer screening (the SiMCerC model) was designed for cervical cancer screening in the Dutch context. The model can simulate a hypothetical population, including cervical cancer progression, and can apply various screening strategies to quantify their effects on the population. The model was coded in C++ at the University of Groningen between 2021 and 2022.

## 2. Description

### *2.1. Model basics*

The SiMCerC model comprises 2 main components: the natural history of cervical cancer and the screening scenarios. Part one is based on the natural history of cervical cancer proposed by Schiffman and Wentzensen. [1] The SiMCerC model has 6 states based on high-risk human papillomavirus (hrHPV), cervical intraepithelial neoplasia (CIN), and cancer status: hrHPV-negative, hrHPV-positive, CIN1, CIN2, CIN3, and cancer (Figure S1).

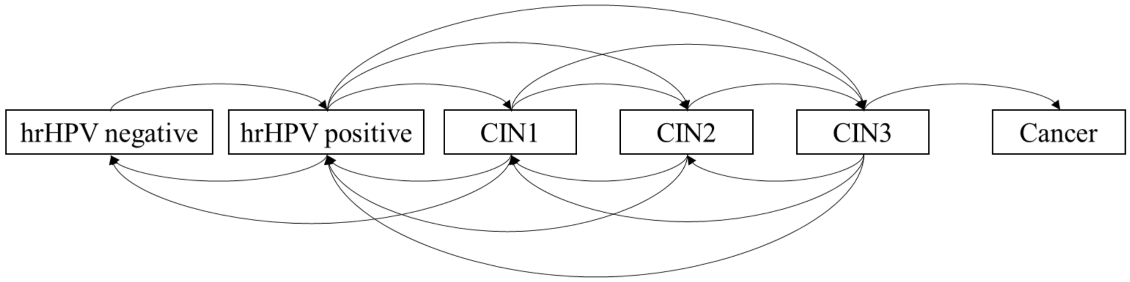


**Figure S1**. The SiMCerC model. Cervical cancer development with consecutive states of hrHPV-negative, hrHPV-positive, CIN1, CIN2, CIN3, and cancer. The arrows represent the possible transition between states. Abbreviations: hrHPV: high-risk human papillomavirus, *CIN: cervical intraepithelial neoplasia.

Once a woman moves to the cancer state, the model can assign a probability of cervical cancer based on the International Federation of Gynecology and Obstetrics (FIGO) stage according to the age-based distribution in the Netherlands for 2018–2019 (Table A1). Additionally, the likelihood of cervical cancer-related death can be calculated using an exponential function of survival, accounting **Table S1. Distribution of FIGO stage probability in patients with cervical cancer by** for age and FIGO stage data from the Netherlands between 2011 and 2019 (Figure S2).

**Table S1. Distribution of FIGO stage probability in patients with cervical cancer by age (95%CI)**

| Age (yrs) | IA | IB | II | III | IV |
|---|---|---|---|---|---|
| <25 | 0% (0.0–52) | 40% (5.2–85) | 60% (15–95) | 0.0% (0.0–52) | 0.0% (0.0–52) |
| 25–34 | 32% (28–38) | 48% (43–54) | 15% (12–20) | 2.3% (1.0–4.6) | 1.7% (0.0–3.2) |
| 35–49 | 27% (24–31) | 43% (39–47) | 20% (17–23) | 4.3% (2.9–6.3) | 6.3% (4.5–8.5) |
| 50–64 | 13% (9.9–17) | 33% (28–38) | 30% (25–35) | 8.0% (5.6–11) | 16% (13–20) |
| 65–74 | 1.8% (0.4–5.3) | 25% (18–32) | 39% (32–47) | 11% (6.7–17) | 23% (17–31) |
| 75+ | 3.2% (1.0–7.3) | 17% (12–24) | 42% (34–50) | 18% (13–25) | 20% (14–26) |

Data retrieved from IKNL (*Integraal Kankercentrum Nederland*, 2022). Abbreviations: CI, confidence interval; FIGO, International Federation of Gynecology and Obstetrics.

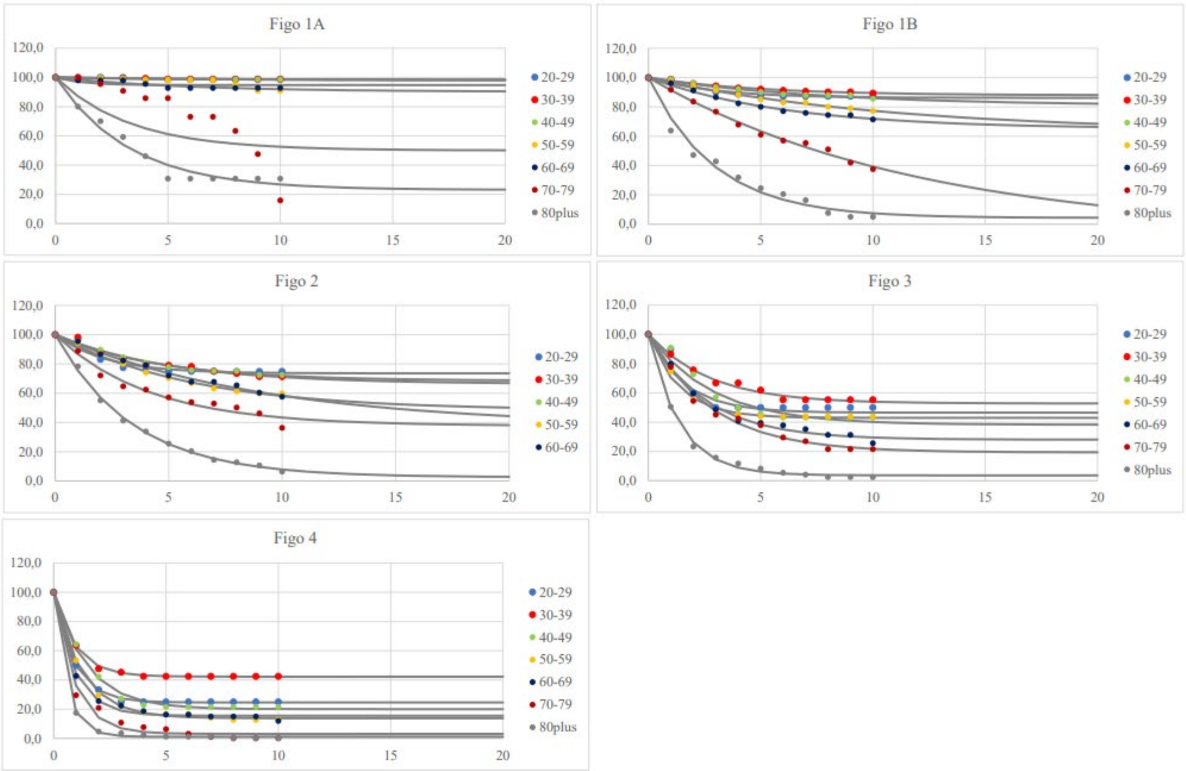


**Figure S2**. Exponential function of cervical cancer survival by age and FIGO. In each graph, a diagnosis of

FIGO stage is depicted. The Y-axis represents survival (%) and the X-axis represents the years after diagnosis. The colors of the dots correspond to different age groups, as indicated in the graph legend.

### *2.2. Transition probabilities*

Transition probabilities between states were obtained from the literature, relying on follow-up studies with absolute or cumulative risk data from one stage to another within specified timeframes (See estimation of transition probabilities section). Priority was given to studies from Dutch populations, followed by European populations, and if unavailable, data from other populations were used. The model simulated a non-HPV-vaccinated cohort from birth to death, considering age-dependent overall death probabilities in the Netherlands.

The following assumptions were applied: 1) progression to CIN1/CIN2/CIN3 states occurs only from an hrHPV-positive state; 2) progression to cervical cancer is only possible from a CIN3 state; 3) there is no direct regression from CIN2/CIN3/cancer states to an hrHPV-negative state; and 4) women cannot live more than 100 years.

## 3. Estimation of transition probabilities

### *3.1. Transition probabilities from hrHPV-negative to hrHPV-positive*

To estimate the transition probabilities from hrHPV-negative to hrHPV-positive, we utilized data from 3 studies reporting hrHPV prevalence by age in Dutch population (Table S2-A4).[2–4] The prevalence data from these studies were aggregated to obtain an overall prevalence per age. However, since prevalence alone is not suitable for estimating transition probabilities, we transformed it into an incidence rate using Equation 1.

**Equation 1:** Incidence rate = prevalence / average duration of disease

We assumed the average duration of hrHPV infection to be 18 months, as reported in Bulkmans et al., 2007.[5] Subsequently, with the incidence rate calculated, we estimated the probability per year using Equation 2 (Table S5).

**Equation 2:** Probability per year = 1 – exp(-rt)

where r is the incidence rate and T is the duration of the cycle (in this case, 1 year).[6]

**Table S2. Prevalence of hrHPV reported by Huijsmans et al., 2016.**

| Age (yrs) | N | hrHPV-positive | Prevalence |
|---|---|---|---|
| 29–33 | 1191 | 223 | 18.7% |
| 34–38 | 1317 | 149 | 11.3% |
| 39–43 | 1775 | 144 | 8.1% |
| 44–48 | 1928 | 135 | 7.0% |
| 49–53 | 1870 | 107 | 5.7% |
| 54–58 | 1703 | 87 | 5.1% |
| 59–63 | 1549 | 65 | 4.2% |

N: indicates the total of women included. hrHPV-positive indicates the number of women who were hrHPV-positive. Abbreviations: hrHPV, high-risk human papillomavirus. Prevalence rates were extrapolated using Dutch data.

**Table S3. Prevalence of hrHPV reported by Lenselink et al., 2008**

| Age (yrs) | N | hrHPV-Positive | Prevalence |
|---|---|---|---|
| 18 | 142 | 9 | 6.3% |
| 19 | 173 | 16 | 9.2% |
| 20 | 190 | 17 | 8.9% |
| 21 | 185 | 26 | 14.1% |
| 22 | 187 | 26 | 13.9% |
| 23 | 185 | 24 | 13.0% |
| 24 | 186 | 26 | 14.0% |
| 25 | 182 | 29 | 15.9% |
| 26 | 186 | 22 | 11.8% |
| 27 | 168 | 34 | 20.2% |
| 28 | 172 | 19 | 11.0% |
| 29 | 109 | 13 | 11.9% |

N: indicates the total of women included. hrHPV-positive indicates the number of women who were hrHPV-positive. Abbreviations: hrHPV, high-risk human papillomavirus. Prevalence rates were extrapolated using Dutch data.

**Table S4. Prevalence of hrHPV reported by Franceschi et al., 2006.**

| Age (yrs) | N | Positive | Prevalence |
|---|---|---|---|
| 15–24 | 26 | 3 | 11.5% |
| 25–34 | 432 | 36 | 8.3% |
| 35–44 | 994 | 24 | 2.4% |
| 45–54 | 1138 | 31 | 2.7% |
| ≥55 | 709 | 12 | 1.7% |

N: indicates the total of women included. hrHPV-positive indicates the number of women who were hrHPV-positive. Prevalence rates were extrapolated using Dutch data.

**Table S5. Transition probability from hrHPV-negative to hrHPV-positive.**

| Age (yrs) | Point estimate | | Lower bound | | Upper bound | |
|---|---|---|---|---|---|---|
| | Incidence rate | P-year | Incidence rate | P-year | Incidence rate | P-year |
| 15–24 | 0.0064 | 0.0737 | 0.0054 | 0.0629 | 0.0074 | 0.0845 |
| 25–34 | 0.0086 | 0.0976 | 0.0078 | 0.0889 | 0.0094 | 0.1061 |
| 35–44 | 0.0043 | 0.0503 | 0.0038 | 0.0451 | 0.0048 | 0.0555 |
| 45–54 | 0.0031 | 0.0361 | 0.0027 | 0.0320 | 0.0034 | 0.0402 |
| ≥55 | 0.0023 | 0.0272 | 0.0020 | 0.0231 | 0.0026 | 0.0312 |

The Incidence was estimated using Equation 1. P-year is the transition probability per year calculated with Equation 2. The lower and upper bounds represent the 95% confidence interval. Abbreviations: hrHPV, high-risk human papillomavirus.

### *3.2. Transition probabilities from hrHPV-positive to hrHPV-negative*

We utilized type-specific hrHPV clearance rates estimated over an 18-month period from the Population-Based Screening Amsterdam trial (POBASCAM).[7] Negative test results for hrHPV type in the follow-up smear over the total for hrHPV type were defined as clearance.[7] To calculate the rate, we transformed the proportion rate into a rate using Equation 3.

**Equation 3:** $r = \{[-\ln(1 - p)] / t\}$

where p is the proportion and t is the time over which the proportion rate was estimated.

With the rate calculated, we used Equation 2 to estimate the probability per year (Table S6). [6]

**Table S6. Transition probability per year from hrHPV-positive to hrHPV-negative.**

| hrHPV-positive to hrHPV-negative | Probability in 18 months | Transition probability per year |
|---|---|---|
| Probability | 0.65 | 0.5034 |
| Lower bound | 0.6 | 0.4571 |
| Upper bound | 0.69 | 0.5420 |

The lower and upper bounds represented the 95% confidence interval. Abbreviations: hrHPV, high-risk human papillomavirus.

### *3.3. Transition probabilities from hrHPV-positive to CIN1*

During model development, we encountered a lack of publications in the Netherlands or Europe studying the progression of hrHPV-positive to CIN1 status. However, we did find a study in China that investigated the progression from hrHPV prevalent types to CIN1. This study included 277

hrHPV-positive women at baseline, of which 117 developed CIN1 within a maximum follow-up period of 63 months (approximately 5.25 years).[8] To ensure accuracy and to account for possible undetected prevalent CIN1 cases, we only considered CIN1 cases detected after 12 months (n = 78).[8] We then applied Equations 2 and 3 to these data (Table S7).[6]

**Table S7. Transition probability from hrHPV-positive to CIN1.**

| hrHPV-positive to CIN1 | Probability in 63 months | Transition probability per year |
|---|---|---|
| Probability | 0.282 | 0.0610 |
| Lower bound | 0.229 | 0.0482 |
| Upper bound | 0.335 | 0.0746 |

The lower and upper bounds represented the 95% confidence interval. Abbreviations: CIN, Cervical intraepithelial neoplasia; hrHPV, high-risk human papillomavirus.

### *3.4. Transition probabilities from hrHPV-positive to CIN2 and CIN3*

We used published data from the POBASCAM trial to estimate transition probabilities.[9] Specifically, we focused on all hrHPV-positive, cytology-negative women (n = 717) who developed either CIN2 (n = 33) or CIN3 (n = 69) during a maximum follow-up period of 14 years.[9] We then applied Equations 2 and 3 to these data (Tables A8-A9).[6]

**Table S8. Transition probability from hrHPV-positive to CIN2.**

| hrHPV-positive to CIN2 | Probability in 14 years | Transition probability per year |
|---|---|---|
| Probability | 0.046 | 0.0034 |
| Lower bound | 0.031 | 0.0022 |
| Upper bound | 0.061 | 0.0045 |

The lower and upper bounds represented the 95% confidence interval. Abbreviations: CIN, Cervical intraepithelial neoplasia; hrHPV, high-risk human papillomavirus.

**Table S9. Transition probability from hrHPV-positive to CIN3.**

| hrHPV-positive to CIN3 | Probability in 14 years | Transition probability per year |
|---|---|---|
| Probability | 0.096 | 0.0072 |
| Lower bound | 0.075 | 0.0055 |
| Upper bound | 0.118 | 0.0089 |

The lower and upper bounds represented the 95% confidence interval. Abbreviations: CIN, Cervical intraepithelial neoplasia; hrHPV, high-risk human papillomavirus.

### *3.5. Transition probabilities from CIN1 to hrHPV-negative, hrHPV-positive, CIN2, and CIN3*

In this section, we used data from Zielinski et al., 2001.[10]

#### 3.5.1. *CIN1 to hrHPV-negative*

The 1-year probability of regression from CIN1 to hrHPV-negative status was reported as the cumulative 1-year incidence of cytological regression, and the regression of abnormal cervical cytology was defined as the presence of normal cytology in 2 consecutive cervical smears.[10] The study focused on women with borderline and mild dyskaryosis (BMD), which can be considered analogous to low-grade squamous intraepithelial lesion (LSIL) in the Bethesda Classification. We used BMD as a proxy for CIN1. The estimated 1-year probability of regression was 40% (95% CI: 26–54).[10]

*3.5.2. CIN1 to hrHPV-positive*

We employed the concept of cumulative 1-year hrHPV persistence,[10] which refers to the absence of viral clearance during follow-up. This persistence was used as the 1-year probability of transitioning from CIN1 to hrHPV-positive status. The study focused on women with a BMD hrHPV+ at baseline, and who remained hrHPV+ throughout follow-up and with no indication of cytological progression. The estimated 1-year regression probability in this context was 16% (95% CI: 6–26)[10].

3.5.3. *CIN1 to CIN2/CIN3*

To estimate the 1-year probability of progression from CIN1 to CIN2, we took the 100 women with BMD who were hrHPV-positive and eligible for follow-up.[10] Among these, 10 women developed CIN2, and 2 women progressed to CIN3 during a follow-up period of 4.3 years.[10] We then applied Equations 2 and 3 to these data (Tables A10-A11).[6]

**Table S10. Transition probability from CIN1 to CIN2.**

| CIN1 to CIN2 | Probability in 4.3 years | Transition probability per year |
|---|---|---|
| Probability | 0.10 | 0.0242 |
| Lower bound | 0.041 | 0.0097 |
| Upper bound | 0.158 | 0.0394 |

The lower and upper bounds represented the 95% confidence interval. Abbreviations: CIN, Cervical intraepithelial neoplasia.

**Table S11. Transition probability from CIN1 to CIN3.**

| CIN1 to CIN3 | Probability in 4.3 years | Transition probability per year |
|---|---|---|
| Probability | 0.020 | 0.0047 |
| Lower bound | 0.000 | 0 |
| Upper bound | 0.047 | 0.0112 |

The lower and upper bounds represented the 95% confidence interval. Abbreviations: CIN, Cervical intraepithelial neoplasia

***3.6. Transition probabilities from CIN2 to hrHPV-positive, CIN1, and CIN3***

To determine all CIN2 progression and regression probabilities, we referred to the study by Nobbenhuis et al., 1999, which had a maximum follow-up of 6 years.[11] The probability of transitioning from CIN2 to CIN1 was estimated by focusing on women who were hrHPV-positive at baseline and had a colposcopy result suggestive of CIN2 (n = 87). Among them, 12 women regressed to CIN1 on histology.[11] To estimate the 1-year probability of transitioning from CIN2 to CIN3, we considered women who were hrHPV-positive at baseline and had a colposcopy suggestive of CIN2 (n = 87). Among them, 22 women showed clinical progression to CIN3 on histology during follow-up.[11] To estimate the 1-year probability of transitioning from CIN2 to hrHPV-positive state, we included all hrHPV-positive women with mild-to-moderate dyskaryosis and severe dyskaryosis (an analog for high-grade squamous intraepithelial lesion (HSIL), as a proxy for CIN2; n = 233) at baseline, from among the 10 hrHPV-persistent women who had CIN0 found during the follow-up period.[11]

We then applied Equations 2 and 3 to these data (Tables A12-A14).[6]

**Table S12. Transition probability from CIN2 to CIN1.**

| **CIN2 to CIN1** | **Probability in 6 years** | **Transition probability per year** |
|---|---|---|
| Probability | 0.138 | 0.0244 |
| Lower bound | 0.065 | 0.0112 |
| Upper bound | 0.210 | 0.0386 |

The lower and upper bounds represented the 95% confidence interval. Abbreviations: CIN, Cervical intraepithelial neoplasia.

**Table S13. Transition probability from CIN2 to hrHPV-positive.**

| **CIN2 to hrHPV-positive** | **Probability in 6 years** | **Transition probability per year** |
|---|---|---|
| Probability | 0.043 | 0.0073 |
| Lower bound | 0.017 | 0.0028 |
| Upper bound | 0.069 | 0.0118 |

The lower and upper bounds represented the 95% confidence interval. Abbreviations: CIN, Cervical intraepithelial neoplasia; hrHPV, high-risk human papillomavirus.

**Table S14. Transition probability from CIN2 to CIN3.**

| **CIN2 to CIN3** | **Probability in 6 years** | **Transition probability per year** |
|---|---|---|
| Probability | 0.253 | 0.0474 |
| Lower bound | 0.162 | 0.0289 |
| Upper bound | 0.344 | 0.0679 |

The lower and upper bounds represented the 95% confidence interval. Abbreviations: CIN, Cervical intraepithelial neoplasia.

### *3.7. Transition probabilities from CIN3 to hrHPV+, CIN1, and CIN2*

To estimate the regression rate from CIN3, we referred to a study conducted in Germany that focused

on spontaneous regression.[12] This retrospective study compared CIN3 biopsy diagnoses with subsequent treatment diagnoses over a maximum follow-up of 66.1 weeks. Among the 635 cases initially diagnosed as CIN3 by biopsy, 39 were later diagnosed during treatment as CIN2, 6 were diagnosed as CIN1, and 3 were categorized as normal (we considered these cases hrHPV-positive since no HPV test was performed during follow-up).[12] We then applied Equations 2 and 3 to these data (Tables A15-A17).[6]

**Table S15. Transition probability from CIN3 to CIN2**.

| CIN3 to CIN2 | Probability in 66.1 weeks | Transition probability per year |
|---|---|---|
| Probability | 0.061 | 0.0486 |
| Lower bound | 0.042 | 0.0338 |
| Upper bound | 0.080 | 0.0636 |

The lower and upper bounds represented the 95% confidence interval. Abbreviations: CIN, Cervical intraepithelial neoplasia.

**Table S16. Transition probability from CIN3 to CIN1.**

| CIN3 to CIN1 | Probability in 66.1 weeks | Transition probability per year |
|---|---|---|
| Probability | 0.009 | 0.0074 |
| Lower bound | 0.019 | 0.0015 |
| Upper bound | 0.017 | 0.0134 |

The lower and upper bounds represented the 95% confidence interval. Abbreviations: CIN, Cervical intraepithelial neoplasia.

**Table S17. Transition probability from CIN3 to hrHPV-positive.**

| CIN3 to hrHPV-positive | Probability in 66.1 weeks | Transition probability per year |
|---|---|---|
| Probability | 0.003 | 0.0025 |
| Lower bound | 0 | 0.000 |
| Upper bound | 0.008 | 0.0059 |

The lower and upper bounds represented the 95% confidence interval. Abbreviations: CIN, Cervical intraepithelial neoplasia; hrHPV, high-risk human papillomavirus

### *3.8. Transition probabilities from CIN3 to Cancer*

Since no reliable study was found to estimate the progression from CIN3 to cancer, we used the published transition probabilities used by Myers et al., 2000, in their Markov model and transformed the data to transition probability per year.[13]

## 4. Cervical cancer screening scenarios

The model also allows for various screening scenarios, generating outcomes for both “no screening” (average life years) and “any screening” (number in primary screening for GP-based sampling and self-sampling, colposcopy referrals, screen-detected CIN2+, and false positives over a lifetime). We made the following assumptions in all screening scenarios:

- All CIN2+ cases detected through screening are treated and subsequently return to the hrHPV-negative state.
- Women who receive treatment for the cancer state will no longer be at risk of acquiring new hrHPV infections or developing new CIN lesions. They will no longer be invited for screening.

The framework for executing the Dutch cervical cancer screening program (2021), which relies on cytology for triage, was used to simulate the base screening scenario.[14] Two different scenarios were then simulated to investigate the effects of molecular testing as an alternative for cytological triage:

- Scenario I: a molecular test was applied to all hrHPV-positive women, regardless of the screening method.
- Scenario II: a molecular test was only applied to hrHPV-positive women who used the self-sampling device.

The flowcharts for the base scenario, scenario I, and scenario II are presented in Figures A3, A4, and A5, respectively.

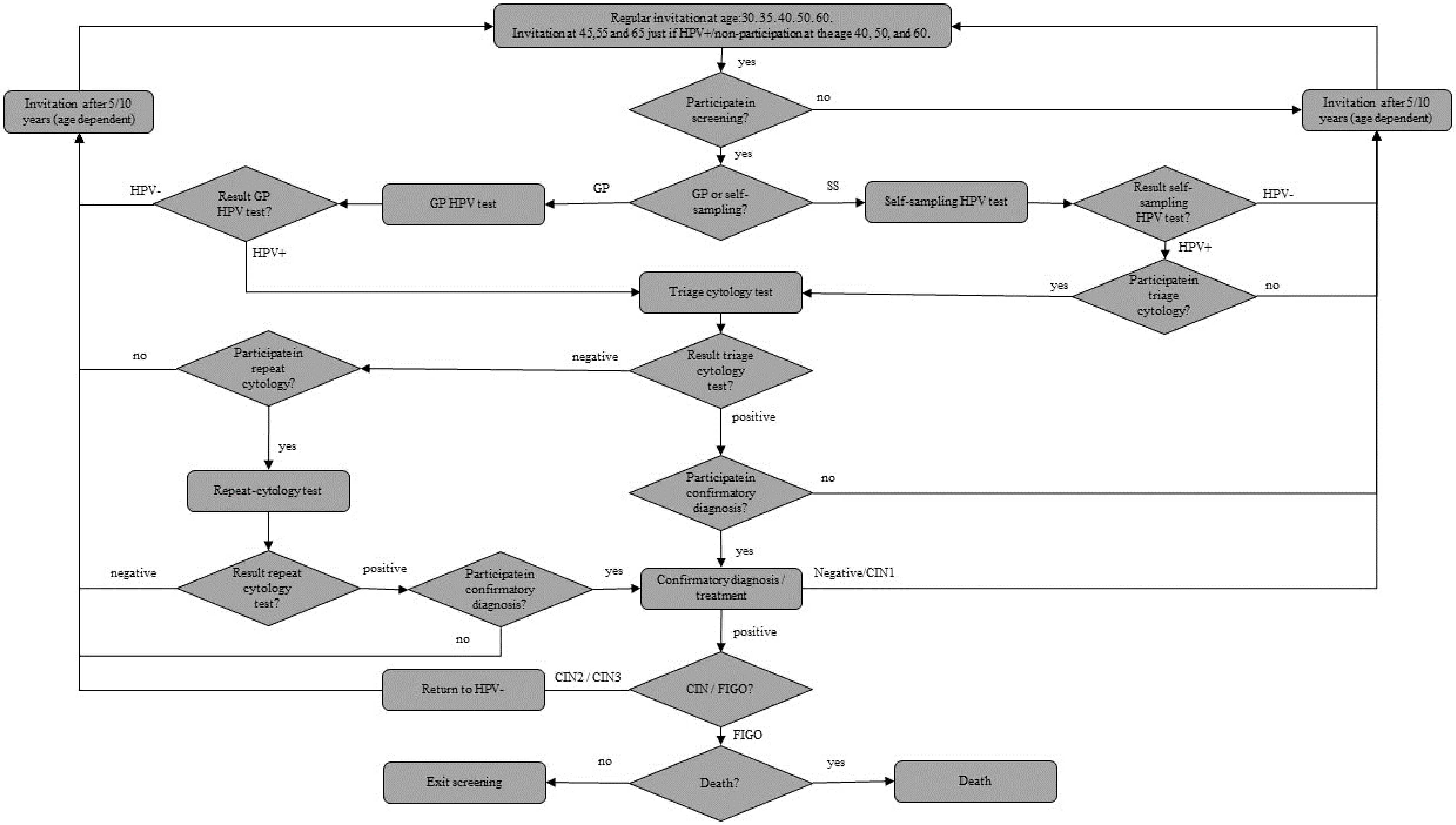


**Figure S3.** Base screening scenario. The framework for the execution of the Dutch cervical cancer screening program (2021), which uses cytology for triage, was used to simulate the base screening scenario. Abbreviations: CIN, Cervical intraepithelial neoplasia; FIGO, International Federation of Gynecology and Obstetrics; GP, general practitioner; hrHPV, high-risk human papillomavirus; SS, self-sampling.

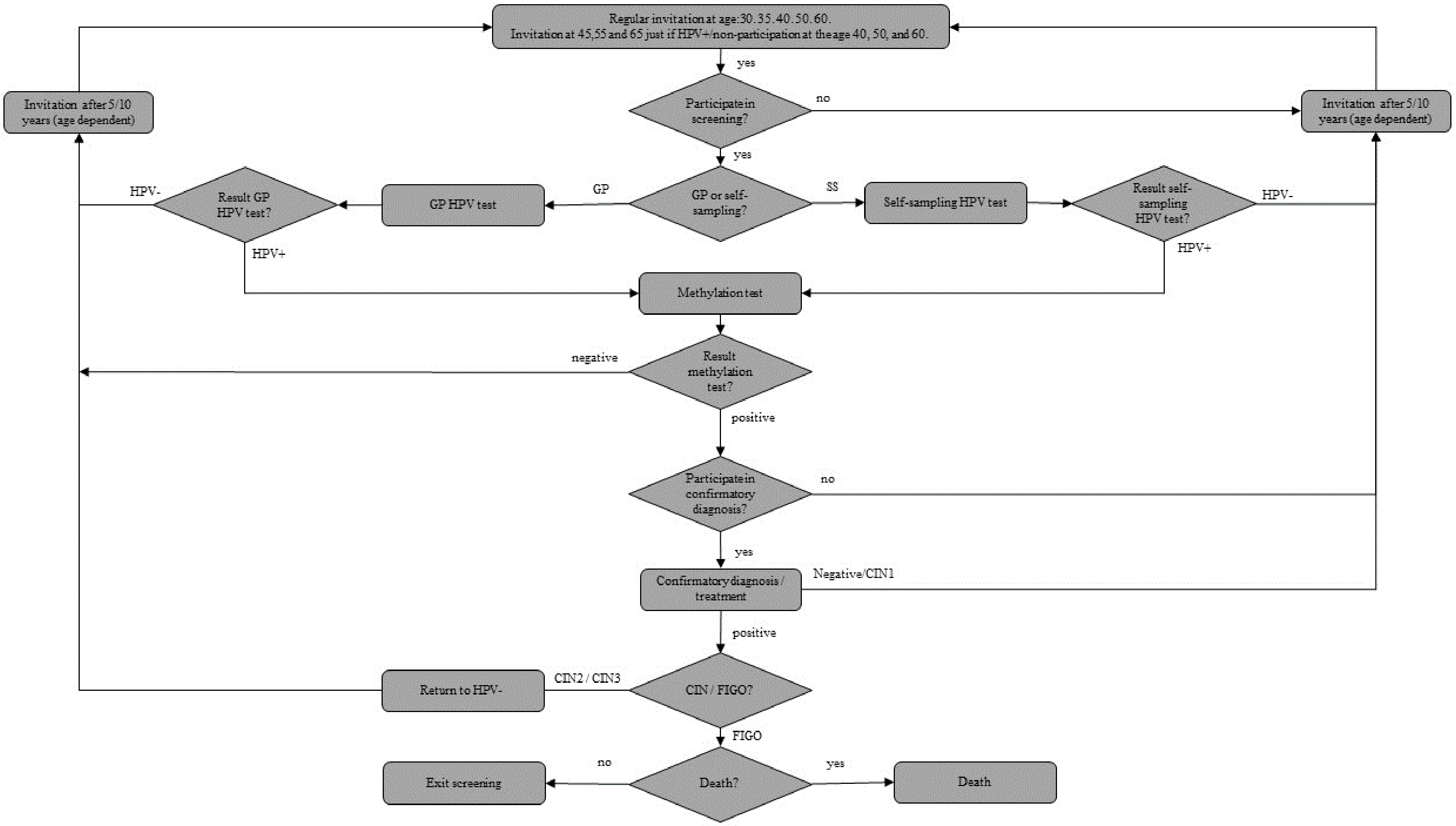


**Figure S4.** Alternative screening scenario I. A molecular test was applied to all hrHPV-positive women. Those who tested positive for the molecular test were referred for confirmatory colposcopy diagnosis, while those who tested negative returned to screening in 5 or 10 years, depending on their age. Abbreviations: CIN, Cervical intraepithelial neoplasia; FIGO, International Federation of Gynecology and Obstetrics; GP, general practitioner; hrHPV, high-risk human papillomavirus; SS, self-sampling.

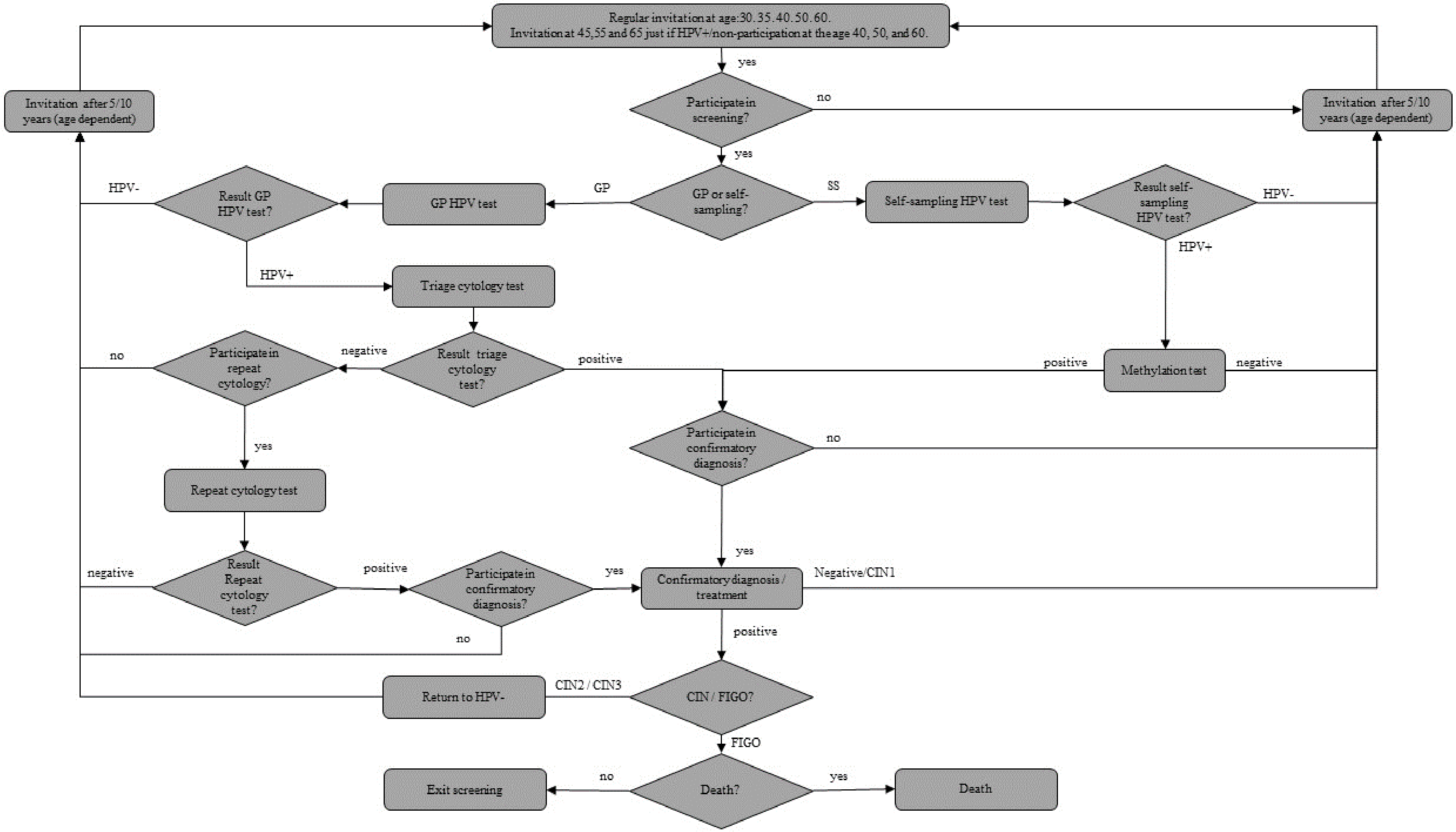


**Figure S5.** Alternative screening scenario II. A molecular test was only applied to hrHPV-positive women who used the self-sampling device, with follow-up the same as in scenario I. Women who underwent hrHPV testing by a GP followed the procedure in the base scenario. Abbreviations: CIN, cervical intraepithelial neoplasia; FIGO, International Federation of Gynecology and Obstetrics; GP, general practitioner; hrHPV, high-risk human papillomavirus; SS, self-sampling.

## Model Validation

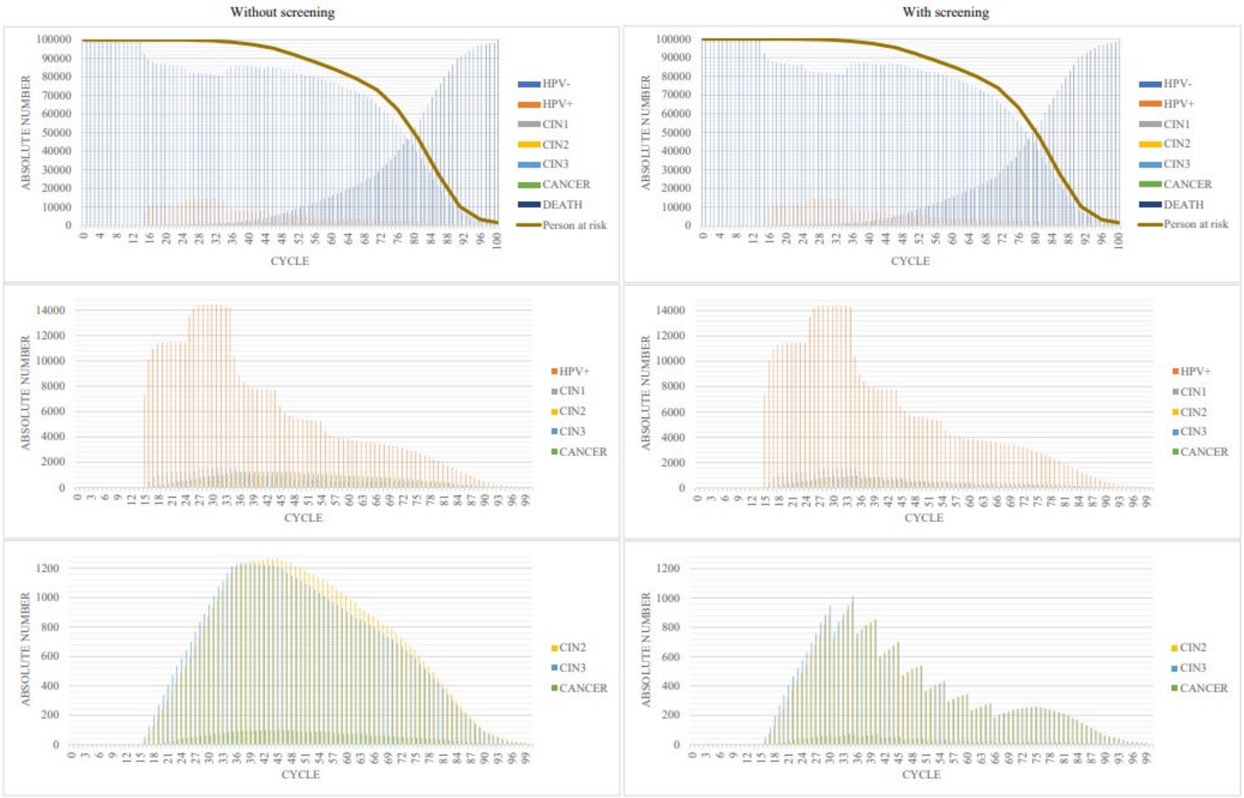


**Figure S6.** Internal validation showing the number of women per state per cycle by screening state.

The prevalence of model states over 100 cycles per year is shown, with the right and left sides representing the simulation with and without screening, respectively. The first row shows the results for the 100,000 simulated women, with the yellow line indicating the number of individuals alive in each cycle. The second row shows a magnified view that allows us to observe the prevalence of hrHPV (in orange) and CIN1 (in gray). The third row provides another magnified view that focuses on the prevalence of CIN2, CIN3, and cancer states per cycle. Abbreviations: CIN, Cervical intraepithelial neoplasia; hrHPV, high-risk human papillomavirus.

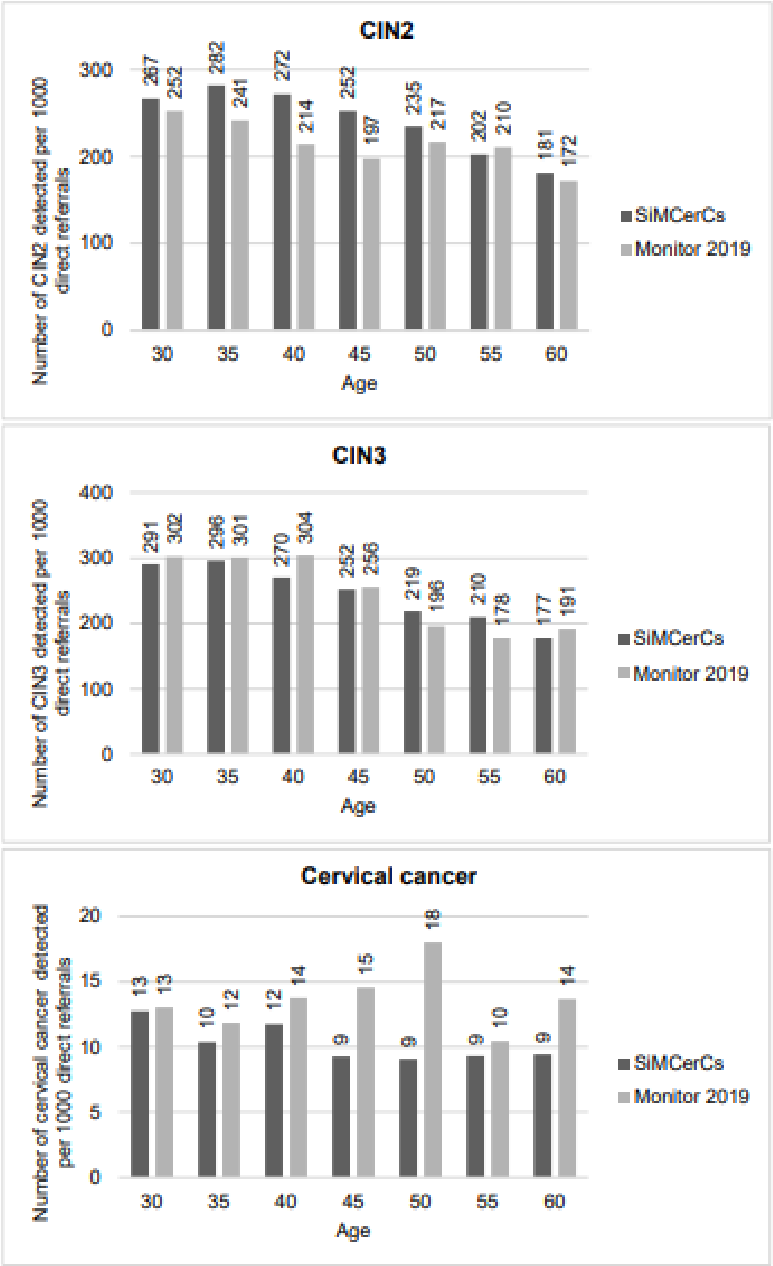


**Figure S7.** Comparison between the SiMCerCs and Monitor 2019 data for the absolute number of CIN2, CIN3, and cancers cases detected.

To allow comparison of the absolute number of CIN2, CIN3, and cancers detected, we normalized the SiMCerCs data and Monitor 2019 data per 1000 direct referrals per age. Abbreviations: CIN, Cervical intraepithelial neoplasia; SiMCerC, a microsimulation Markov model for cervical cancer screening.

**References**


1. Schiffman M, Wentzensen N. Human papillomavirus infection and the multistage carcinogenesis of cervical cancer. *Cancer Epidemiol Biomarkers Prev [Internet]* 2013;22:553–60. Available from: https://www.ncbi.nlm.nih.gov/pubmed/23549399
2. Huijsmans CJ, Geurts-Giele WR, Leeijen C, Hazenberg HL, van Beek J, de Wild C, van der Linden JC, van den Brule AJ. HPV Prevalence in the Dutch cervical cancer screening population (DuSC study): HPV testing using automated HC2, cobas and Aptima workflows. *BMC Cancer [Internet]* 2016;16:922. Available from: https://www.ncbi.nlm.nih.gov/pubmed/27894291
3. Lenselink CH, Melchers WJ, Quint WG, Hoebers AM, Hendriks JC, Massuger LF, Bekkers RL. Sexual behaviour and HPV infections in 18 to 29 year old women in the pre-vaccine era in the Netherlands. *BMJ [Internet]* 2012;3:e3743. Available from: https://www.ncbi.nlm.nih.gov/pubmed/19011683
4. Franceschi S, Herrero R, Clifford GM, Snijders PJ, Arslan A, Anh PT, Bosch FX, Ferreccio C, Hieu NT, Lazcano-Ponce E, Matos E, Molano M, et al. Variations in the age-specific curves of human papillomavirus prevalence in women worldwide. *Int J Cancer [Internet]* 2006;119:2677–84. Available from: https://www.ncbi.nlm.nih.gov/pubmed/16991121
5. Bulkmans NW, Berkhof J, Bulk S, Bleeker MC, van Kemenade FJ, Rozendaal L, Snijders PJ, Meijer CJ, Group PS. High-risk HPV type-specific clearance rates in cervical screening. *Br J Cancer [Internet]* 2007;96:1419–24. Available from: https://www.ncbi.nlm.nih.gov/pubmed/17342094
6. Gidwani R, Russell LB. Estimating Transition Probabilities from Published Evidence: A Tutorial for Decision Modelers. *Pharmacoeconomics [Internet]* 2020;38:1153–64. Available from: https://doi.org/10.1007/s40273-020-00937-z
7. Bulkmans NW, Berkhof J, Bulk S, Bleeker MC, van Kemenade FJ, Rozendaal L, Snijders PJ, Meijer CJ, Group PS. High-risk HPV type-specific clearance rates in cervical screening. *Br J Cancer [Internet]* 2007;96:1419–24. Available from: https://www.ncbi.nlm.nih.gov/pubmed/17342094

8. Lazare C, Xiao S, Meng Y, Wang C, Li W, Wang Y, Chen G, Wei J, Hu J, Xue M, Wu P. Evaluation of Cervical Intraepithelial Neoplasia Occurrence Following the Recorded Onset of Persistent High-Risk Human Papillomavirus Infection: A Retrospective Study on Infection Duration. *Front Oncol* 2019;9:976.

9. Dick S, Kremer WW, De Strooper LMA, Lissenberg-Witte BI, Steenbergen RDM, Meijer CJLM, Berkhof J, Heideman DAM. Long-term CIN3+ risk of HPV positive women after triage with FAM19A4/miR124-2 methylation analysis. *Gynecol Oncol* 2019;154:368–73.

10. Denise Zielinski G, Snijders PJ, Rozendaal L, Voorhorst FJ, Runsink AP, de Schipper FA, Meijer CJ. High-risk HPV testing in women with borderline and mild dyskaryosis: long-term follow-up data and clinical relevance. *J Pathol* 2001;195:300–6.

11. Nobbenhuis MA, Walboomers JM, Helmerhorst TJ, Rozendaal L, Remmink AJ, Risse EK, van der Linden HC, Voorhorst FJ, Kenemans P, Meijer CJ. Relation of human papillomavirus status to cervical lesions and consequences for cervical-cancer screening: a prospective study. *Lancet* 1999;354:20–5.

12. Motamedi M, Bohmer G, Neumann HH, von Wasielewski R. CIN III lesions and regression: retrospective analysis of 635 cases. *BMC Infect Dis [Internet]* 2015;15:541. Available from: https://www.ncbi.nlm.nih.gov/pubmed/26589896

13. Myers ER, McCrory DC, Nanda K, Bastian L, Matchar DB. Mathematical model for the natural history of human papillomavirus infection and cervical carcinogenesis. *Am J Epidemiol* 2000;151:1158–71.

14. National Institute for Public Health and the Environment. Framework for the execution of the Dutch cervical cancer screening programme [Internet]. 2021. Available from: https://www.rivm.nl/documenten/framework-for-execution-of-cervical-cancer-population-screening.
